# *Staphylococcus aureus* encodes four differentially regulated pyruvate transporters

**DOI:** 10.1128/jb.00163-25

**Published:** 2025-10-10

**Authors:** Jennifer L. Endres, Cleofes Sarmiento, William Xiao, Marat R. Sadykov, Kenneth W. Bayles, McKenzie K. Lehman

**Affiliations:** 1 Department of Pathology, Microbiology, and Immunology, University of Nebraska Medical Center 12284, Omaha, Nebraska, USA; 2 Department of Genetics, Cell Biology and Anatomy, University of Nebraska Medical Center 12284, Omaha, Nebraska, USA; University of Illinois Chicago, Chicago, Illinois, USA

**Keywords:** pyruvate, transport, metabolism, lactate

## Abstract

The success of *Staphylococcus aureus* as a pathogen is attributable, in part, to its ability to exploit the diverse nutrient sources available during infection. Critical to this success are the pathways involving pyruvate that serve as a nexus for energy production, oxidative metabolism, and biosynthetic processes. When available, bacteria acquire pyruvate from the environment to fuel growth. Recently, LrgAB was identified as a pyruvate transporter under microaerobic conditions, leading us to speculate that *S. aureus* encodes other pyruvate transporters that are active during aerobic growth. In this study, we used the toxic pyruvate analog, 3-fluoropyruvic acid (3-FP), to isolate mutants with impaired pyruvate uptake. Whole-genome sequencing (WGS) of these mutants revealed mutations in two genes, *lctP* and *lldP*. Pyruvate uptake was significantly delayed when both *lctP* and *lldP* were inactivated. Although LldP and LctP were annotated as L-lactate permeases, ^14^C-pyruvate uptake assays confirmed that they function as pyruvate transporters. Despite a reduction in pyruvate uptake, the *lctP lldP* mutant did not have a growth defect in media with pyruvate, indicating that there may be an additional pyruvate importer. Reassessment of 3-FP susceptibility of the *lctP lldP* mutant revealed a zone of inhibition, confirming there is another transporter. WGS of 3-FP-resistant *lldP lctP* mutants identified B7H15_13955, an annotated MFS transporter, as the fourth transporter. Importantly, inactivation of all four genes completely eliminated pyruvate uptake, suggesting we have identified all the pyruvate transporters. These findings reveal that *S. aureus* employs multiple pyruvate transporters to support pyruvate metabolism under aerobic and anaerobic conditions.

**IMPORTANCE:**

Pyruvate is a key metabolite that supports bacterial energy production in many conditions. While the LrgAB system was previously implicated in pyruvate import under microaerobic conditions, the transporters that enable *Staphylococcus aureus* pyruvate acquisition during aerobic growth have remained undefined. We identified *lctP* and *lldP*, two genes annotated as lactate transporters, and B7H15_13955 as additional pyruvate transporters. Through genetic inactivation, pyruvate consumption, growth, and ^14^C-pyruvate uptake assays, we demonstrate that LctP, LldP, and B7H15_13955 are capable of pyruvate import, and with LrgAB, comprise a regulated network for pyruvate acquisition. This discovery fills a critical gap in our understanding of *S. aureus* metabolic adaptation and reveals that this pathogen is equipped with multiple systems to import pyruvate under diverse environmental conditions.

## INTRODUCTION

Pyruvate metabolism is a critical determinant of bacterial fitness and viability, as it plays a pivotal role in maintaining cellular homeostasis and energy production under aerobic and anaerobic conditions. It is a key intermediate of central metabolism and plays a crucial role as a carbon distribution point for oxidative metabolism, overflow metabolite production, and fatty acid and amino acid biosynthesis (1–3). As a key intermediate in a variety of metabolic pathways, the acquisition of this molecule from the surrounding environment serves as an important alternative energy source.

Most of the studies of prokaryotic pyruvate import have been focused on the model organism, *Escherichia coli*, which encodes three pyruvate transporters, including an inducible, high-affinity pyruvate/H^+^ symporter, BtsT (4), a constitutive pyruvate transporter, CstA (5), and YhjX (6). CstA was initially characterized as a peptide transporter until further investigations demonstrated that *cstA* inactivation resulted in decreased pyruvate uptake (7). Moreover, studies have demonstrated that the inactivation of *btsT* resulted in decreased pyruvate uptake (8). As expected, inactivation of both *cstA* and *btsT* resulted in an even more significant decrease in pyruvate uptake (8), demonstrating the additive effect of these transporters in acquiring this metabolite.

While best known for its role as a cell death effector, recent studies of the *lrgAB* operon in several bacterial species have revealed its involvement in pyruvate uptake (9–11), suggesting that the Cid/Lrg family of proteins plays an uncharacterized role in metabolism centered on the pyruvate node. In *Staphylococcus aureus*, disruption of the *lrgAB* operon caused a significant growth defect under low-oxygen conditions when grown with pyruvate as the primary carbon source, which correlated with decreased pyruvate consumption (10). Given that the growth of the *lrgAB* mutant was comparable to the parental strain under aerobic conditions with pyruvate as the carbon source, we hypothesized that *S. aureus* encodes an additional transporter(s) capable of mediating pyruvate uptake under these conditions (10).

In this study, we identified three additional genes (*lctP*, *lldP,* and B7H15_13955) involved in *S. aureus* pyruvate uptake. Although annotated as lactate transporters, we were able to demonstrate a role for LctP and LldP in pyruvate import, but not lactate, during aerobic growth using ^14^C-pyruvate uptake assays. Furthermore, our results demonstrate the redundancy and differential regulation of these transporters, with the presence of B7H15_13955 as a pyruvate transporter revealed in a secondary suppressor screen with regulatory data indicating carbon catabolite repression of this newly identified pyruvate importer.

## RESULTS

### Inactivating mutations in *lctP* and *lldP* confer resistance to a toxic pyruvate analog

**Fig 1:**
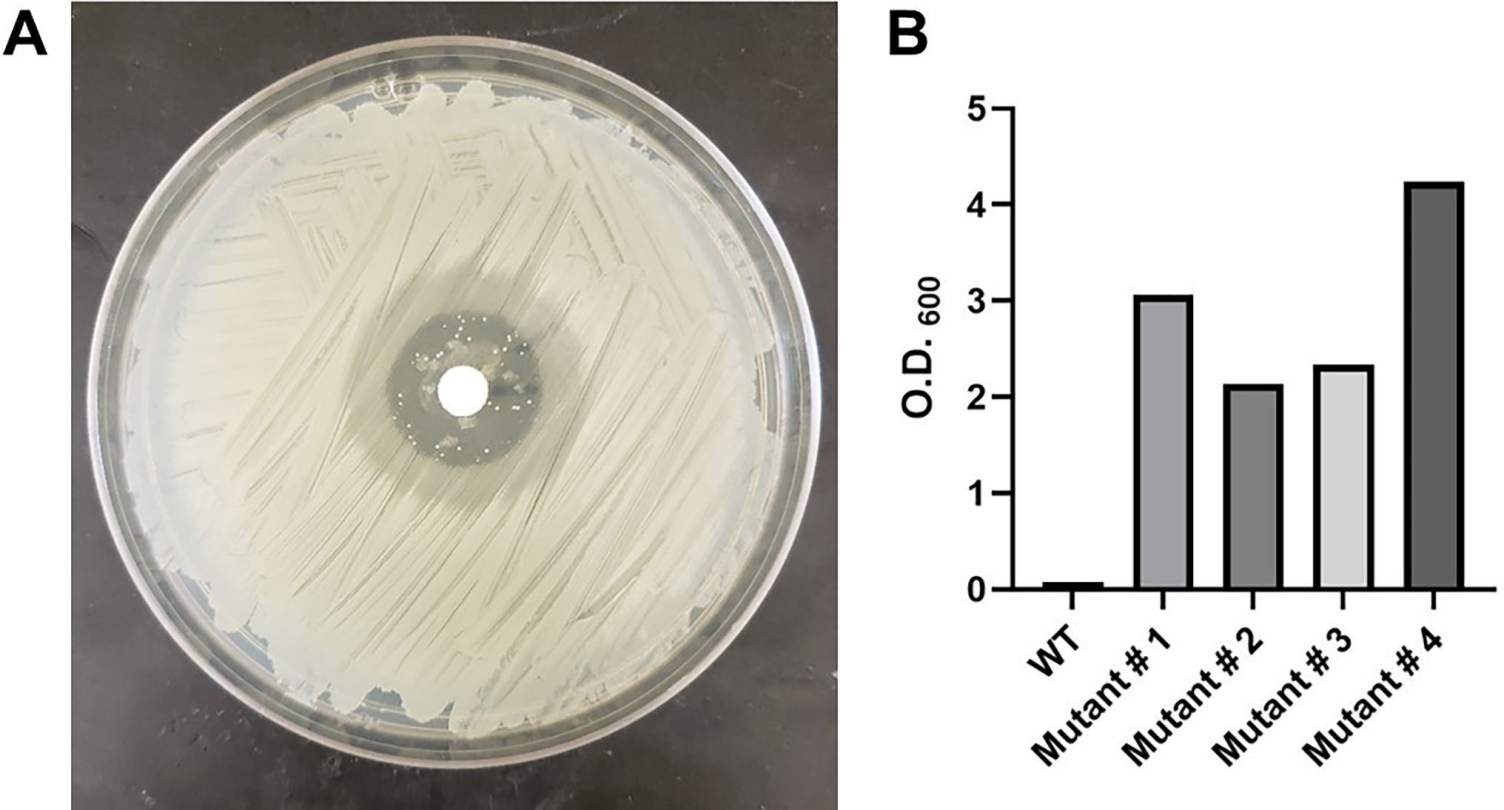
Isolation of 3-FP-resistant mutants. (**A**) The toxic pyruvic acid analog 3-FP was placed on a disk in the center of a lawn of *S. aureus* JE2 on TSA . After 24 hours of growth at 37°C, resistant colonies were observed in the large zone of inhibition. (**B**) Growth yield of JE2 and 3-FP mutants grown in 5 mL TSBG + 3 mM 3-FP overnight at 37°C.

Recent studies revealed that the *lrgAB* operon plays an important role in the acquisition of pyruvate under microaerobic conditions when pyruvate is the primary carbon source (10). To identify genes involved in the acquisition of pyruvate under aerobic conditions, we utilized a toxic pyruvate analog, 3-fluoropyruvic acid (3-FP) (12), to select for mutations in genes, such as pyruvate-specific transporters, that confer resistance to 3-FP. The *S. aureus* laboratory strain, JE2, was spread on the surface of tryptic soy agar (TSA) plates, and then disks saturated with 3-FP were placed in the center of each plate. After overnight incubation at 37°C, colonies that emerged in the resulting clearing zones (Fig. 1A) were isolated and subsequently tested for resistance to 3-FP. After confirming 3-FP resistance in liquid culture (Fig. 1B), four JE2 mutants were subjected to whole-genome sequencing (WGS) to identify any mutations that were potentially associated with the resistance phenotype. As shown in Table 1, each of the resistant strains had a frameshift mutation in the putative L-lactate permease, *lctP*, which resulted in a truncated protein product of 301 amino acids versus the wildtype at 530 amino acids. Furthermore, three of the four resistant strains had unique mutations that resulted in the inactivation of a second putative L-lactate permease, *lldP*, indicating that *S. aureus* may encode two additional pyruvate transporters.

**TABLE 1:** Mutations identified via WGS of 3-FP-resistant mutants isolated from JE2^*a*^

Strain	Locus	Gene, Gene Product	Mutation	Amino acid change
JE2 M1	B7H15_00615	*lctP,* L-lactate permease	A→ -	Frameshift (301)
JE2 M2	B7H15_00615	*lctP,* L-lactate permease	A→ -	Frameshift (301)
	B7H15_04795	2-hydroxyacid dehydrogenase	G→A	ala→thr
	B7H15_13125	*lldP,* L-lactate permease	C→T	gln→stop (112)
JE2 M3	B7H15_00615	*lctP,* L-lactate permease	A→ -	Frameshift (301)
	B7H15_13125	*lldP,* L-lactate permease	A→ -	Frameshift (159)
JE2 M4	B7H15_00615	*lctP,* L-lactate permease	A→ -	Frameshift (301)
	B7H15_13125	*lldP,* L-lactate permease	A→T	lys→stop (182)

^*a*^ Mutations and loci were identified based on the reference genome of *S. aureus* JE2 (GenBank accession CP020619). The number in parentheses indicates the location of the amino acid in the protein that is mutated. LctP is a 530 amino acid protein, and LldP is a 532 amino acid protein.

### LctP and LldP are important for pyruvate consumption during aerobic growth

**Fig 2:**
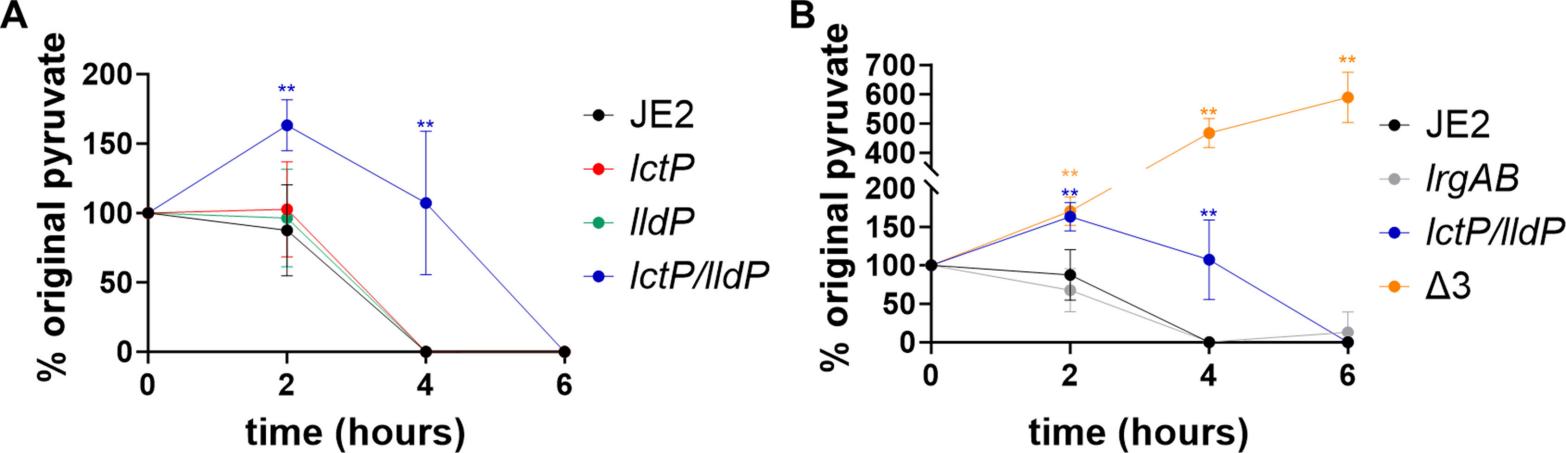
Pyruvate consumption is reduced in the *lctP/lldP* mutant. *S. aureus* JE2 and isogenic mutants (**A**) *lctP*, *lldP*, and *lctP*/*lldP* or (**B**) *lrgAB*, *lctP/lldP*, and *lctP/lldP/lrgAB (*Δ3) were grown in TSBG with 0.5 mM pyruvate for 6 hours. Supernatants were collected every 2 hours and analyzed to determine the amount of pyruvate remaining in the media. The percent original is calculated based on the measured amount (0.5 mM) of pyruvate added to the media after inoculation. Data represent the mean ± SD from three independent experiments, each with three technical replicates. A two-way ANOVA determined statistical significance compared to wildtype represented by **=*P* < 0.005.

Because we found multiple 3-FP-resistant strains with mutations in both *lldP* and *lctP*, we constructed a double mutant (*lctP/lldP*) to determine if these genes have redundant functions. To assess the impact of these mutations on pyruvate import, we measured the concentration of exogenous pyruvate during growth. To accomplish this, we grew JE2 and the putative pyruvate mutant strains in standard TSB that contains 0.25% glucose (14 mM), here referenced as TSBG, supplemented with pyruvate (0.5 mM) and determined the extracellular concentration of pyruvate as described by Endres et al. (10). Inactivation of *lctP* and *lldP* alone caused a minimal but reproducible decrease in depletion of exogenous pyruvate compared to the wild-type strain, but inactivation of both *lctP* and *lldP* resulted in delayed pyruvate depletion (Fig. 2A). Additionally, we introduced the *lrgAB* mutation into the *lctP/lldP* mutant, generating a triple mutant (Δ3) with all of the putative transporters inactivated (Fig. 2B). Interestingly, the triple mutant exhibited an increased concentration of pyruvate in the medium after 4 and 6 hours of growth, suggesting that it likely cannot import pyruvate and may instead be exporting pyruvate into the media. Together, these data indicate that LldP and LctP are important for pyruvate uptake from the media. Furthermore, the observation that the triple mutant has 5× higher pyruvate at later phases of growth suggests that the import and export of pyruvate is mediated by separate transporters.

### The *lctP* and *lldP* genes encode pyruvate transporters

**Fig 3:**
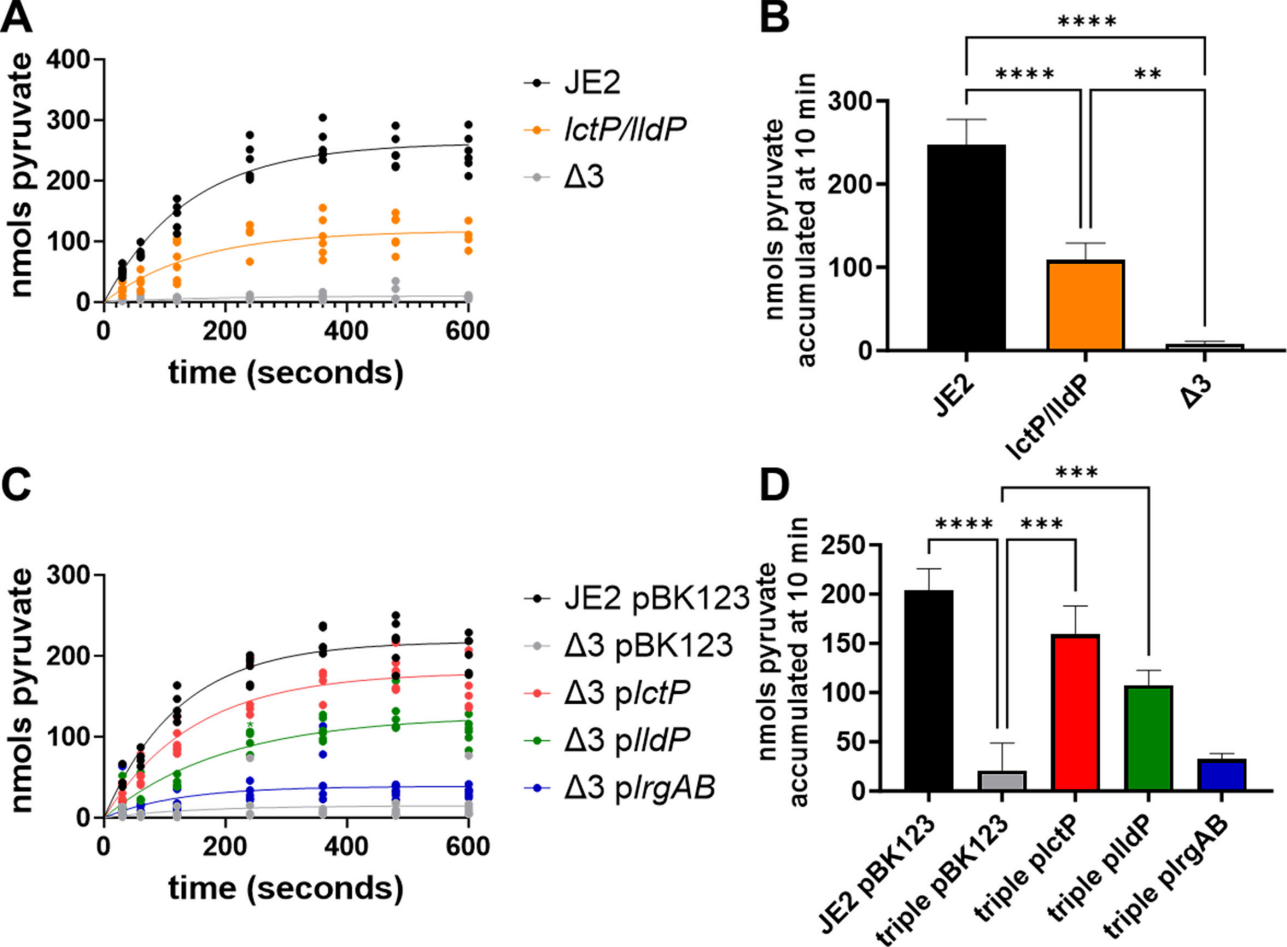
LctP, LldP, and LrgAB transport pyruvate. ^14^C-pyruvate transport assays and total nmols pyruvate accumulated at 10 minutes of pyruvate transporter mutants including (**A**) and (**B**) JE2, *lctP/lldP*, and *lctP/lldP/lrgAB* (Δ3) or (**C**) and (**D**) Δ3 strains complemented with either the empty cadmium inducible expression vector (pBK123) or the vector with pyruvate transporter (p*lctP,* p*lldP*, and p*lrgAB*). The stains were grown in TSBG with 0.5 mM pyruvate for 4 hours prior to the uptake assay. All ^14^C-pyruvate transport assay data fit by nonlinear regression using a first-order equation of technical duplicates ran three independent times. The total accumulated data represent mean ± SD of the 10 minutes timepoint. Brown-Forsythe and Welch ANOVA tests, with Dunnett’s T3 multiple comparisons, were applied to determine statistical significance compared to either (**B**) the respective strains or (**D**) Δ3 pBK123 with **=*P* < 0.005, ***=*P* < 0.0005, and ****=*P* < 0.0001.

We observed significantly elevated exogenous pyruvate levels in the *lctP/lldP* and Δ3 mutants (Fig. 2), suggesting these three proteins impact pyruvate import. To provide direct evidence that LctP, LldP, and/or LrgAB are capable of transporting pyruvate, we utilized ^14^C-pyruvate uptake assays to assess the import of this metabolite into the cells. Briefly, the strains were grown for four hours in TSBG containing 0.5 mM pyruvate, the same conditions used to monitor exogenous pyruvate levels in Fig. 2. The cells were washed, and approximately 10^8^ cells were incubated with ^14^C-pyruvate at a final concentration of 0.5 mM pyruvate, with samples taken over a 10-minute time course. As shown in Fig. 3A and B, the level of pyruvate associated with the *lctP lldP* double mutant was significantly lower compared with the wild-type strain. However, the Δ3 strain in which all three loci were disrupted resulted in nearly undetectable levels of ^14^C-pyruvate taken up by the cells, indicating its ability to transport pyruvate into the cell is almost completely abrogated under these conditions. To determine the relative contributions of the three pyruvate transporters, we assessed ^14^C-pyruvate uptake in the Δ3 mutant complemented individually with each putative pyruvate transporter under the control of a cadmium-inducible promoter. The results of these experiments indicate that all three of the putative pyruvate transporters contribute to pyruvate import, with *lctP* playing the most significant role under these conditions, followed by *lldP*, and then *lrgAB* (Fig. 3C and D).

**Fig 4:**
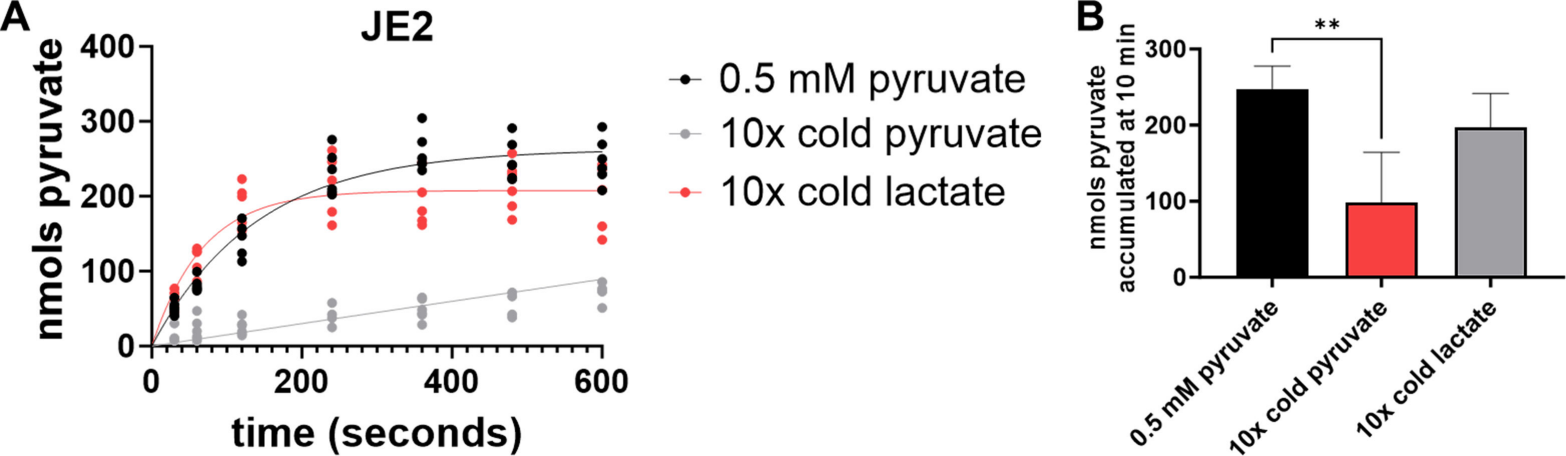
LctP and LldP are pyruvate-specific transporters. (**A**) ^14^C-pyruvate transport assays and (**B**) total nmols pyruvate accumulated at 10 minutes of JE2 with 0.5 mM ^14^C-pyruvate or excess (10×, 5 mM) unlabeled lactate or pyruvate. The stains were grown in TSBG with 0.5 mM pyruvate for 4 hours prior to the uptake assay. All ^14^C-pyruvate transport assay data fit by nonlinear regression using a first-order equation of technical duplicates ran three independent times. Total accumulated data represent mean ± SD of the 10 minutes timepoint. Brown-Forsythe and Welch ANOVA tests, with Dunnett’s T3 multiple comparisons, were applied to determine statistical significance compared to JE2 with 0.5 mM pyruvate with **=*P* < 0.005.

Because *lctP* and *lldP* were annotated as lactate permeases, we sought to assess the specificity of these transporters for pyruvate. To do so, we performed the same ^14^C-pyruvate uptake experiment using the wild-type JE2 strain in the presence of excess (5 mM) cold pyruvate or lactate. As anticipated, the presence of cold pyruvate significantly reduced the uptake of the ^14^C-pyruvate (Fig. 4). In contrast, the presence of unlabeled excess lactate had a minimal effect on the uptake of the labeled pyruvate (Fig. 4), indicating that the transporters encoded by these three pyruvate transport loci are not significant contributors to lactate uptake under the conditions tested.

### *S. aureus* encodes a fourth pyruvate transporter

With the significant decrease in ^14^C-pyruvate import in the triple mutant (Fig. 3A and B), we hypothesized that we would observe decreased growth yield aerobically in media supplemented with pyruvate as an additional carbon source. Aerobic growth curves were performed in chemically defined media (CDM) supplemented with either glucose (CDMG; 14 mM), pyruvate (CDMP; 28 mM), or lactate (CDML; 28 mM). Surprisingly, under all conditions tested, we did not observe a decrease in growth yield when compared to the wild-type strain, despite observing a notable decrease in growth yield in all of the strains in CDM not supplemented with an additional carbon source (Fig. S1). This suggests that either the other carbon sources (i.e., amino acids) present in the culture medium may have masked the potential impact of these mutations on growth, or there is an additional pyruvate transporter capable of importing pyruvate under these conditions.

**Fig 5:**
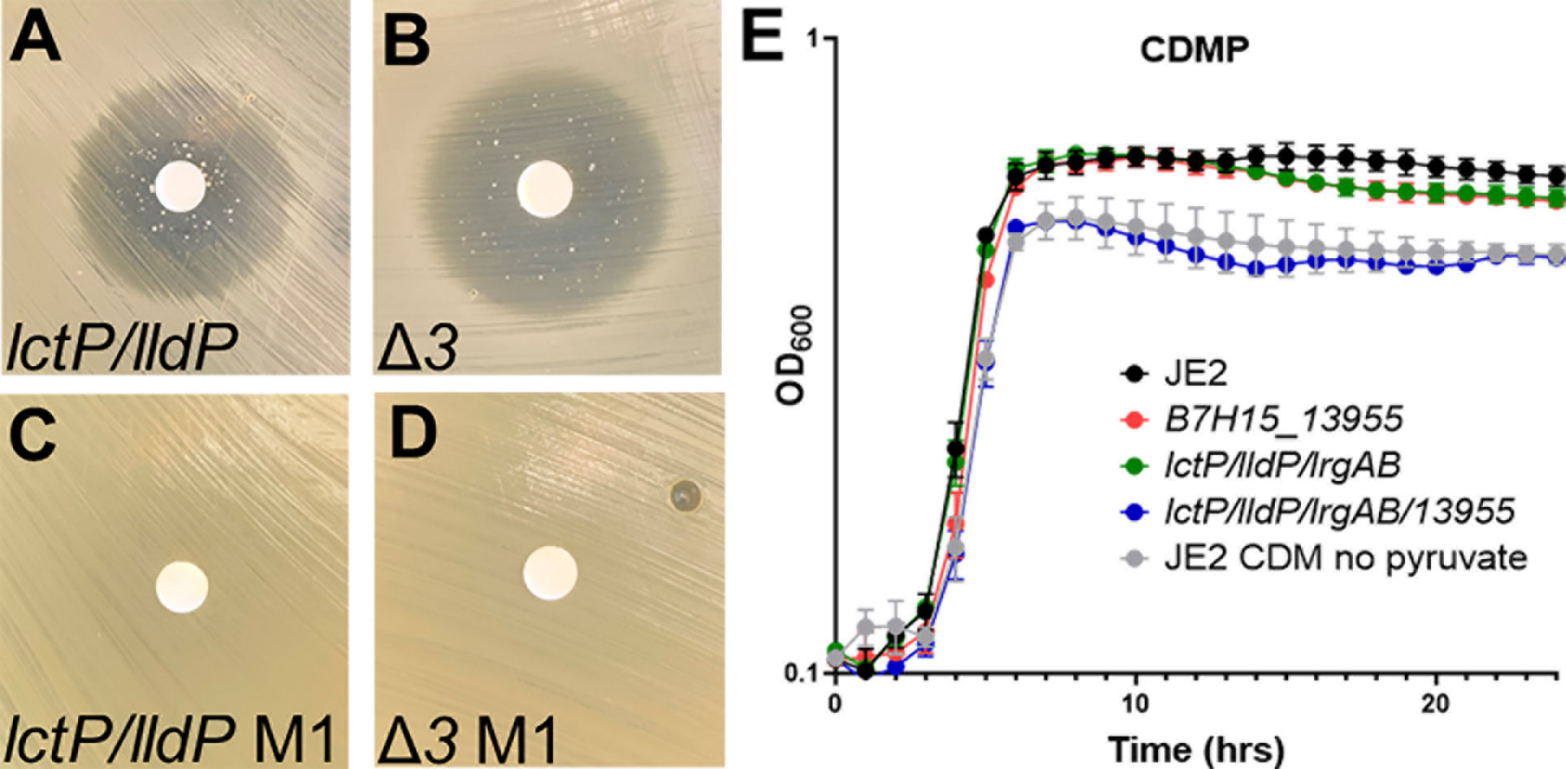
B7H15_13955 encodes a fourth pyruvate transporter. The toxic pyruvic acid analog 3-FP was placed on a disk in the center of a lawn of (**A**) *lctP/lldP* or (**B**) Δ3 on TSA. After 24 hours of growth at 37°C, resistant colonies were observed in the large zone of inhibition. Suppressor mutants isolated from the zone of inhibition of the (**C**) *lctP/lldP* and (**D**) Δ*3* mutants were confirmed to have full resistance to 3-FP. (**E**) Aerobic growth analysis of JE2 in CDM and CDM with 28 mM pyruvate (CDMP) and the following mutants: B7H15_13955, *lctP/lldP/lrgAB*, *lctP/lldP/lrgAB/13955* in CDMP. Data represent the mean ± SD from three biological experiments, repeated three times.

To test the hypothesis that there is an additional transporter, we sought to confirm that the *lldP/lctP* and Δ3 mutant were indeed fully resistant to the toxic pyruvate analog, 3-FP. Surprisingly, we observed a zone of inhibition (Fig. 5A), albeit smaller than that observed in the wild-type strain, when a disk saturated with 3-FP was placed on a lawn of *lctP/lldP* grown on TSBG (Table S1), indicating there was another transporter. We also tested the susceptibility of the Δ3 mutant to 3-FP and found that it too had a zone of inhibition (Fig. 5B), although slightly hazy, supporting the hypothesis that there was indeed an additional, fourth pyruvate transporter. From the zone of inhibition, we isolated individual colonies that were growing near the 3-FP saturated disk of the *lctP/lldP* and Δ3 mutant. We confirmed that these colonies were 3-FP resistant by observing growth up to the 3-FP saturated disk (Fig. 5C and D). WGS of two 3-FP-resistant *lctP/lldP* strains and one Δ3 strain revealed unique SNPs or inactivating deletions in an annotated MFS transporter (B7H15_13955; Table 2).

**TABLE 2:** Mutations identified via WGS of 3-FP-resistant *lctP/lldP* or *lctP/lldP/lrgAB* (Δ3) mutants isolated from JE2^*a*^

Strain	Locus	Gene, Gene Product	Mutation	Amino acid change
*lctP/lldP* M1	B7H15_13955	MFS transporter	Deletion	Deletion
*lctP/lldP* M2	B7H15_06085	*argF*, ornithine carbamoyltransferase	A→T	Asn → Tyr (170)
	B7H15_13955	MFS transporter	C→T	Trp → stop (10)
*lrgAB/lctP/lldP* (Δ3) M1	B7H15_03795	hypothetical protein	- →A	stop (21)
	B7H15_05165	hypothetical protein	G→C	Ile → Met (16)
	B7H15_08260	aldo/keto reductase	T→-	stop (282)
	B7H15_13265	GAF domain-containing protein	-→T	stop (26)
	B7H15_13605	*opuCa*, glycine/betaine ABC transporter ATP-binding protein	C→T	Glu → Lys (383)
	B7H15_13955	MFS transporter	C→T	Trp → stop (367)

^*a*^ Mutations and loci were identified based on the reference genome of *S. aureus* JE2 (GenBank accession CP020619). The number in parentheses indicates the location of the amino acid in the protein that is mutated. B7H15_13955 encodes a protein 425 aa long.

### *S. aureus* encodes four pyruvate transporters

Because we observed unique mutations in B7H15_13955 in the three sequenced 3-FP-resistant strains, we introduced the B7H15_13955 mutant into the Δ3 strain, generating a quadruple mutant (Δ4; *lctP/lctP/lrgAB/13955*) and assessed growth in CDM supplemented with pyruvate. The Δ4 mutant exhibited a reduced growth yield in CDM supplemented with pyruvate, similar to the growth of the wild-type strain grown in CDM (Fig. 5E). These data suggest that we have identified all of the pyruvate transporters encoded by *S. aureus*.

**Fig 6:**
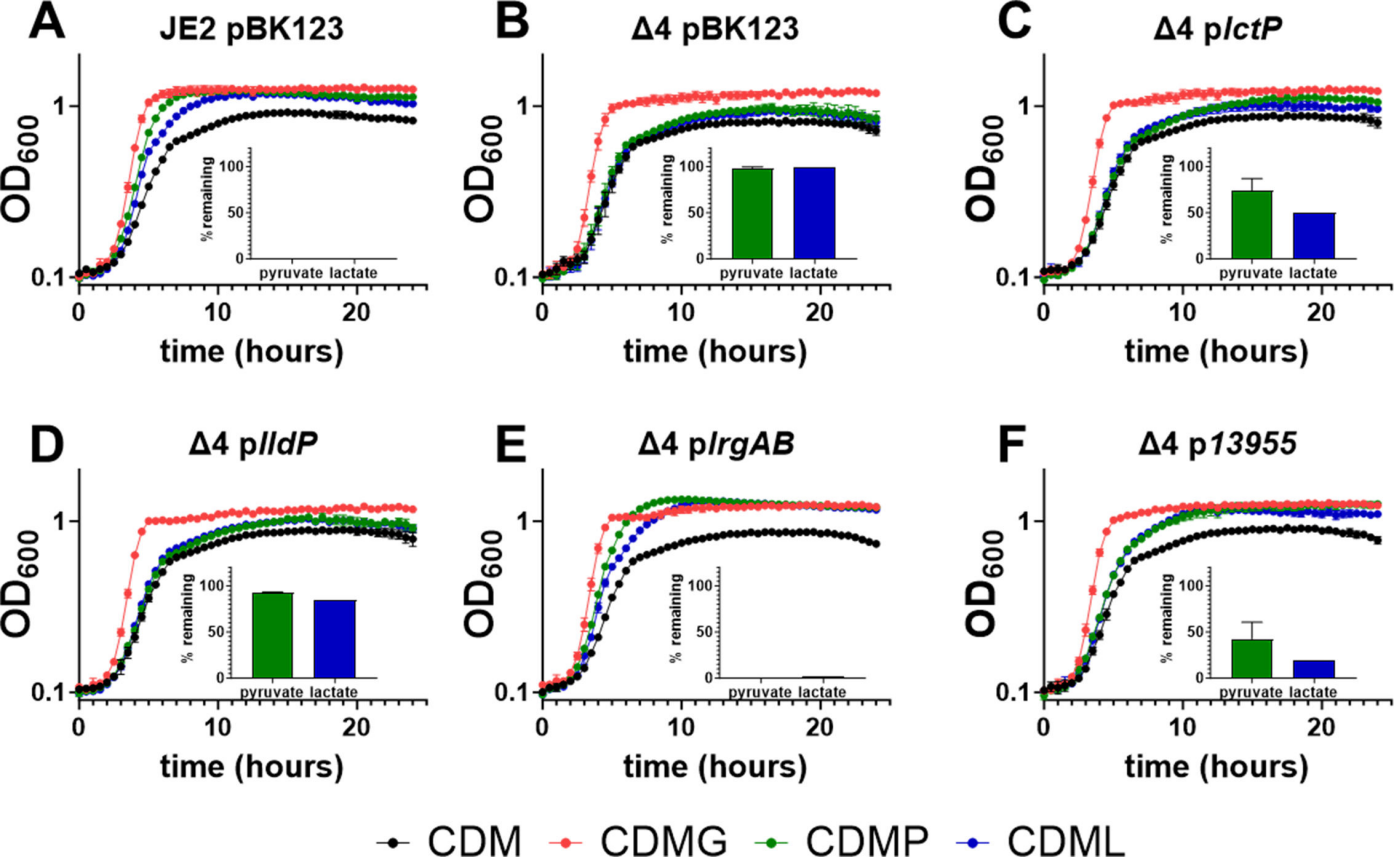
B7H15_13955 and LrgAB are capable of pyruvate and lactate import. Aerobic growth analysis of (**A**) JE2 pBK123, (**B**) Δ4 pBK123, (**C**) Δ4 p*lctP*, (**D**) Δ4 p*lldP*, (**E**) Δ4 p*lrgAB*, and (**F**) Δ4 p*13955* in CDM, CDM with 14 mM glucose (CDMG), CDM with 28 mM pyruvate (CDMP), and CDM with 28 mM lactate (CDML). Bar graph inserts are exogenous pyruvate and lactate measurements taken 24 hours after the growth curve was completed, with the % remaining calculated based on the amount measured in the medium prior to inoculating with bacteria. Data represent the mean ± SD from three biological experiments, repeated three times.

To determine the function of each of the individual transporters, we utilized complementation vectors with the expression driven by a cadmium-inducible promoter as were used for *lctP*, *lldP*, and *lrgAB* expression in Fig. 3C and D. Under aerobic conditions, we observed that the Δ4 mutant with the empty vector (pBK123) produced a decreased growth yield in CDM supplemented with pyruvate (CDMP) or lactate (CDML), similar to the growth yield observed in CDM (Fig. 6B), suggesting this strain was incapable of utilizing the lactate and pyruvate added to the media. Conversely, there was no difference in growth yield in CDM supplemented with glucose (CDMG) compared to the wild-type strain (Fig. 6A), confirming that the *lctP*, *lldP*, *lrgAB*, and B7H15_13955 genes did not affect glucose metabolism. Interestingly, overexpression of B7H15_13955 and *lrgAB* resulted in full restoration of the Δ4 mutant growth yield in CDM supplemented with lactate and pyruvate (Fig. 6E and F), indicating these two transporters are likely monocarboxylate transporters capable of importing both lactate and pyruvate. Overexpression of *lctP* resulted in a slight enhancement of growth in both pyruvate and lactate (Fig. 6C) but not to the levels observed in B7H15_13955 and *lrgAB* complementation. Lastly, overexpression of *lldP* resulted in a minor enhancement of growth in the presence of either pyruvate or lactate (Fig. 6D). Exogenous pyruvate and lactate measurements at 24 hours correlate with the observed growth phenotypes (Fig. 6), suggesting that it is the ability (or lack thereof) to import pyruvate or lactate that is dictating the growth yield observed in the complemented strains. Overall, these data indicate that B7H15_13955 is a fourth transporter capable of importing pyruvate, and therefore, we named this locus MonT, for monocarboxylate transporter.

### Differential regulation of the pyruvate transporters

**Fig 7:**
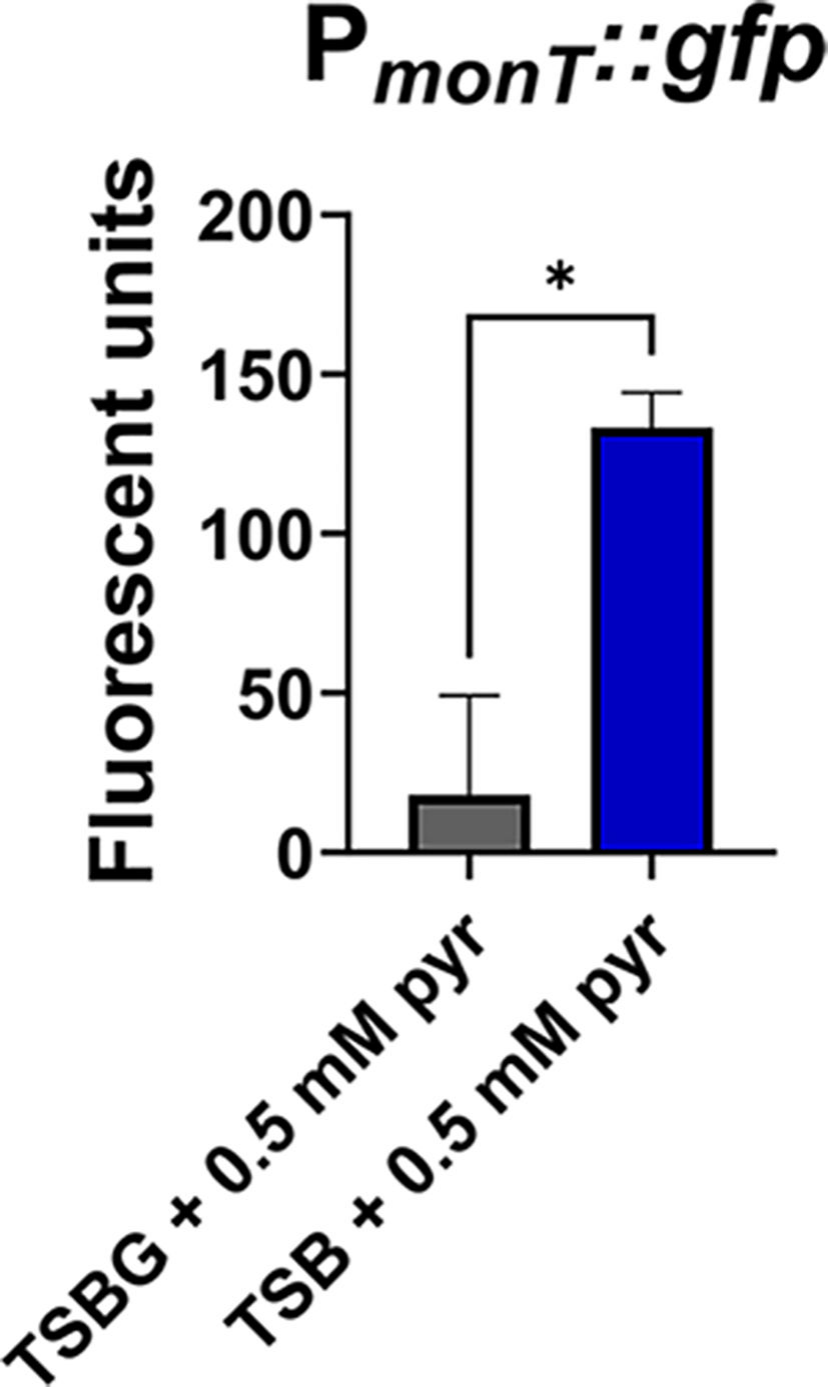
Glucose-mediated repression of *monT*. GFP expression of the wild-type strain, JE2, with P*_monT_::gfp* reporter plasmid, grown aerobically to the exponential phase of growth (4 hours) in TSB or TSBG (14 mM glucose) supplemented with 0.5 mM pyruvate. Data are the representative of the mean of fluorescent units/OD_600_ of three biological replicates ±SD. Welch’s *t*-test was used to determine statistical significance, with *=*P* < 0.05.

The ^14^C-pyruvate transport assays showed a significant reduction in pyruvate import in the Δ3 mutant (Fig. 3), causing us to initially overlook the fourth transporter, MonT. We hypothesized that the ^14^C-pyruvate transport assays were performed in conditions in which expression of *monT* was repressed. Because the assays were performed in TSBG supplemented with 0.5 mM pyruvate, we postulated that *monT* was repressed by the carbon catabolite repressor, CcpA. Bioinformatic analysis revealed a potential catabolite response element site upstream of *monT*. To test that glucose represses *monT* expression, we generated a vector with the *monT* promoter driving the expression of GFP. We monitored GFP expression in TSB or TSBG supplemented with 0.5 mM pyruvate grown aerobically, the latter being the condition in which we performed the initial pyruvate consumption assays (Fig. 2) and ^14^C-pyruvate transport assays (Fig. 3). We observed minimal GFP expression in the P*_monT_::gfp* vector in TSBG + 0.5 mM pyruvate and a statistically significant increase in expression when glucose was removed from the media (Fig. 7), suggesting that *monT* is repressed when glucose is present. Overall, these data indicate that the repertoire of pyruvate transporters is differentially regulated, and there are likely specific conditions in which each of these transporters are expressed and function as a pyruvate and/or lactate importers.

## DISCUSSION

Pyruvate is an important metabolite that feeds the TCA cycle under aerobic conditions or yields byproducts of fermentation (lactate, acetate, etc.) under microaerobic/anaerobic conditions or in the presence of excess glucose (1, 9, 13). Although bacteria have multiple pathways leading to the production of pyruvate to drive downstream metabolic pathways (5, 14, 15), they are also well equipped to assimilate pyruvate from the environment. Indeed, several pyruvate transporters, including those encoded by the *btsT*, *cstA*, and *yhjX* genes, have been identified in organisms such as *E. coli* using the toxic pyruvate analog, 3-FP (8). In *S. aureus*, our laboratory demonstrated a role for the *lrgAB* operon in the assimilation of pyruvate (10), consistent with studies of *lrgAB* homologs in *Streptococcus mutans* and *Bacillus subtilis* (9, 16). However, since *S. aureus lrgAB*-mediated pyruvate assimilation was limited to microaerobic conditions, it seemed likely that *S. aureus* encodes additional pyruvate transporters, as growth was enhanced by the addition of pyruvate during aerobic growth (17). Because bioinformatic approaches failed to identify these additional pyruvate transporter genes, we employed an untargeted genetic approach using 3-FP to generate pyruvate transport mutants. WGS of the mutants generated revealed mutations in three genes, *lctP*, *lldP*, and *monT* (Tables 1 and 2).

Some eukaryotic organisms possess monocarboxylate transporters involved in the proton-dependent transport of L-lactate, pyruvate, short fatty acids, and monocarboxylate drugs in various tissues (18). Also, transporters for pyruvate and lactate can be symporters and antiporters, moving these metabolites either in the same or opposite directions (8, 19, 20). Other organisms separate the transport of pyruvate and lactate and utilize different transporters to import each metabolite, such as the case with *E. coli* lactate transporters *lctP* and *lldP*, and pyruvate transporters *btsT*, *cstA*, and *yhjX* (4, 8, 12, 21). In contrast, the *Shewanella oneidensis lctP2* gene appears to be required for growth when either pyruvate or lactate is used as a carbon source, suggesting it is able to mediate the transport of either metabolite (22). Our ^14^C-pyruvate transport assays with excess cold pyruvate and lactate indicated that under the conditions tested, pyruvate was the primary monocarboxylate transported, as the cold lactate did not reduce ^14^C-pyruvate import, whereas cold pyruvate did (Fig. 4). In contrast, growth curves revealed that the Δ4 mutant had reduced growth in CDM supplemented with either lactate or pyruvate (Fig. 6), which could be fully complemented by overexpression of *lrgAB*, indicating it is likely a monocarboxylate transporter (Fig. 6E). Similarly, *monT* overexpression fully restored growth yield to wild-type levels in CDM supplemented with lactate and pyruvate (Fig. 6F), suggesting it too is a monocarboxylate transporter. Despite significant import of ^14^C-pyruvate in rich media with glucose (Fig. 3), overexpression of either *lctP* or *lldP* only slightly enhanced growth in CDM with pyruvate or lactate. Because the expression of complementation of the pyruvate transporters is under the regulation of the cadmium-inducible promoter, we postulate that other conditions not yet explored are also dictating which monocarboxylate molecule is preferentially imported.

In other bacterial species, the *lctP* and *lldP* genes and their homologs seem to function as lactate transporters. In *B. subtilis,* a *lldP* homolog (51% identity with *S. aureus lldP*), designated *lutP*, is important for growth when lactate is the primary carbon source, acting as the primary transporter for L-lactate import (23). Although disruption of the *B. subtilis lctP* gene did not produce a similar phenotype, it was hypothesized that *lctP* is primarily involved in exporting excess L-lactate generated through glycolysis rather than importing external L-lactate for use as a carbon source. Furthermore, the authors suggested that *lctP* may be specifically adapted for functioning under hypoxic conditions, where lactate dehydrogenase enzymes tend to favor the reduction of pyruvate to lactate, generating excess lactate that needs to be exported. In *S. aureus*, CidC may be performing a similar function, except converting pyruvate to acetate that is ultimately exported (24, 25). In contrast, disruption of the *Azospirillum brasilense lldP* homolog (44% identity with *S. aureus lldP*) had a significant effect on the ability of this organism to grow when either lactate or pyruvate was provided as the sole carbon source (22). Thus, despite the sequence similarity between these genes, the *S. aureus* orthologs appear to have evolved different specificities for lactate and pyruvate.

Interestingly, the *E. coli* two-component regulatory system, BtsSR (formerly YehUT), which regulates the high-affinity transporter BtsT, shares structural similarities with the *S. aureus* LytSR two-component regulatory system, which regulates *lrgAB* expression (4, 26, 27). The BtsT protein (formerly known as YjiY) is a secondary transport protein in *E. coli* that functions as a high-affinity pyruvate/H^+^ symporter (4). Current research suggests that it belongs to the CstA-like transport protein family and is encoded by the *btsT* gene (5, 8). The BtsT transporter becomes vital during the transition from exponential to stationary growth phase, during which cells experience nutrient depletion. The expression of *btsT* is induced in response to extracellular pyruvate (similar to *lrgAB* [10]) and plays a crucial role in being able to scavenge pyruvate under carbon-limited conditions. Typical of transport proteins, BtsT contains multiple transmembrane domains and is believed to use the proton gradient across the membrane to drive pyruvate uptake (4).

Pyruvate transport is a vital process in both eukaryotes and bacteria, playing a crucial role in regulating cellular metabolism and energy production. Despite the differences in cellular organization and complexity, the regulation of pyruvate transport appears to be similar between these two domains of life. In eukaryotes, the mitochondrial pyruvate carrier (MPC) complex, composed of MPC1 and MPC2 proteins, has similar biochemical properties. The MPC1 and MPC2 proteins are small (12 and 15 kDa), transmembrane-containing proteins that form a heteromeric complex within the inner mitochondrial membrane (28). *In vitro* and *in vivo* experiments with these proteins reveal that both MPC1 and MPC2 are necessary for the transport of pyruvate (similar to what is seen with LrgAB) (29). In addition, the three MPC proteins of yeast have been found to form different complexes dependent on whether they are under respiratory or fermentative growth conditions, where MPC1 will either form a complex with MPC2 or MPC3, dependent on its environmental condition (30). Evolutionarily, this segregation may have emerged as a consequence of the trade-offs between competing selective pressures, favoring the development of specialized proteins that function under specific conditions (31). Studies in our laboratory indicate a similar relationship between pyruvate transporters, oxygen levels, and carbon sources may also exist in bacteria. For example, Endres et al. (10) identified a role for the *lrgAB*-encoded proteins in pyruvate transport under microaerobic conditions, suggesting the existence of other pyruvate transporters that were functional during aerobic growth. Indeed, the identification of additional pyruvate transporters that function under aerobic conditions as described in the current study is consistent with this hypothesis.

In summary, pyruvate metabolism is a vital process in both eukaryotes and prokaryotes, playing an important role in regulating cellular metabolism and energy production, ultimately impacting the pathogenicity of the organism (32). Despite the differences in cellular organization and complexity, the mechanisms underlying pyruvate transport exhibit striking similarities between these two domains of life. Current studies are focused on the identification of the conditions in which *S. aureus* benefits from the acquisition of pyruvate from the environment, as well as the role this metabolite may have during pathogenesis. With the extensive number of genes dedicated to pyruvate uptake and their differential regulation, it is likely that the acquisition of pyruvate from unique niches is important for the survival of *S. aureus*.

## MATERIALS AND METHODS

### Bacterial strains, plasmids, and growth conditions

All strains and plasmids used in these studies are listed in Table 3. The *S. aureus* JE2 clean *lrgAB* mutant was generated by allelic exchange utilizing the previously described allelic exchange plasmid, pJB54 (10). Defined *Bursa aurealis* transposon mutants for *lctP::Φ*NE, *lldP::Φ*NE, and *monT::Φ*NE were obtained from the Nebraska Transposon Mutant Library and backcrossed into JE2 using Φ11 (33). In order to generate the double and triple mutants, the erythromycin resistance cassette encoded on the transposon in *lctP::Φ*NE was changed out to a kanamycin resistance cassette (34). This mutation was then moved into the clean Δ*lrgAB* or the Δ*lrgAB/lldp::Φ*NE isolate via Φ11 as previously described (34). To generate the quadruple mutant, the erythromycin resistance cassette encoded on the transposon in *monT::Φ*NE was changed out to the tetracycline resistance cassette and was then moved into the Δ3 via Φ11. Strains were confirmed with primers flanking the gene, or with a gene and transposon (antibiotic cassette) specific primers listed in Table 3. All strains were then evaluated by pulsed-field gel electrophoresis to confirm that no large genome arrangements occurred during genomic manipulations.

**TABLE 3:** List of strains and plasmids used in this study

Bacterial strains	Description	Reference
*E. coli*		
DH5α	Strain used for recombinant plasmid	35
*S. aureus*		
RN4220	Restriction-deficient strain, highly transformable	36
*S. aureus* JE2	Wildtype USA300 isolate	33
JE2 Δ*lrgAB*	JE2 *lrgAB* marker-less mutant	This study
JE2 *lctP::kan*	*lctP::ΦNE* transposon mutant with erythromycin resistance replaced with kanamycin	This study
JE2 *lldP::erm*	*lldP::ΦNE* transposon mutant with erythromycin resistance	This study
JE2 *13955::tet (monT::tet*)	*monT*::*ΦNE* transposon mutant with erythromycin resistance replaced with tetracycline	This study
JE2 *lctP::kan/lldP::erm*	*lctP::kan* transduced into *lldP::erm* via Φ11	This study
JE2 Δ*lrgAB/lldP::erm*	*lldP::erm* transduced into Δ*lrgAB via* Φ11	This study
JE2 Δ*lrgAB/lctP::kan/lldP::erm* (Δ3)	*lctP::kan* transduced into *ΔlrgAB*, *lldP::erm* via Φ11	This study
JE2 Δ*lrgAB/lctP::kan/lldP::erm/monT::tet* (Δ4)	*monT::tet* transduced into Δ3 via Φ11	This study
Plasmids		
pBK123	Empty chloramphenicol resistant derivative of pCN51 shuttle vector that includes the cadmium inducible promoter	37
pMRSII	Dual reporter shuttle vector	38
P_cad_::*lctP*	pBK123 with the *lctP* ORF cloned behind the cadmium inducible promoter at the SalI/EcoRI sites	This study
P_cad_::*lldP*	pBK123 with the *lldP* ORF cloned behind the cadmium inducible promoter at the SalI/EcoRI sites	This study
P_cad_::*lrgAB*	pBK123 with the *lrgAB* operon cloned behind the cadmium inducible promoter at the SalI/EcoRI sites	This study
P_cad_::*13955*	pBK123 with the B7H15_13955 ORF cloned behind the cadmium inducible promoter at the SalI/NdeI sites	This study
pML21	*monT* promoter driving sGFP cloned into the SphI/NdeI sites of pMRSII	This study

The P*_cad_::lctP* (p*lctP*), P*_cad_::lldP* (p*lldP*), P*_cad_::lrgAB* (p*lrgAB*), and P*_cad_::13955* (p*13955*) plasmids were created by amplifying the entire ORF of each gene from JE2 chromosomal DNA with the respective primers listed in Table 2. The amplified fragments were then inserted into the SalI and EcoRI sites of pBK123 directly downstream of the encoded P*_cad_* promoter by Gibson assembly (37, 39). Plasmids were confirmed by sequencing in (35) *E. coli* DH5α, and then electroporated into RN4220 as an intermediary strain (40) prior to transduction into the JE2 Δ3, or JE2 Δ4 isolate via Φ11. Plasmids were retained with 10 µg mL^−1^ chloramphenicol for any pre-growth.

For growth analysis, *S. aureus* strains were grown overnight in standard tryptic soy broth with 14 mM glucose (TSBG) medium (BD Biosciences) at 37°C with aeration at 250  rpm. The overnight cultures were then pelleted, washed, and inoculated to a final OD_600_ of 0.05 in wells of a 96-well plate in the respective media (CDM [41], CDMG [14 mM glucose], CDMP [28 mM pyruvate], or CDML [28 mM lactate]), and aerobic growth was monitored by OD_600_ measurements taken every 30 minutes using a Tecan plate reader. For the extracellular pyruvate analysis in TSBG + 0.5 mM pyruvate (Fig. 2), bacterial cultures were inoculated at an OD_600_ of 0.05 into a 250 mL flask with a 1:10 vol to flask ratio and grown aerobically (shaking, 250 RPM) for 6 hours.

### Generation of GFP promoter fusion

The promoter region of *monT* was amplified from JE2 chromosomal DNA with the respective primers from Table 4. Gibson assembly was used to clone the amplified fragments into pMRSII, a dual reporter shuttle vector carrying both DsRed and GFP, that had been digested with SphI and NdeI removing the DsRed (38). The plasmid was confirmed by sequencing in (35) *E. coli* DH5α and then electroporated into RN4220 as an intermediary strain (40) prior to transduction into the JE2 wild type via Φ11. Plasmids were retained with 10 µg mL^−1^ chloramphenicol.

**TABLE 4:** Oligonucleotides used in this study

Primers	Sequences	Description
1	GGTGTCAAGATGCAAGTTGGAC	lrgAB-F
2	CCCAGTTATAAACTGGAGTATAGACG	lrgAB-R
3	CGATTGCCTGTCACATATAGGAG	lctP-F
4	GACAAGATGCTCATTGCATTTCG	lctP-R
5	GCCAGAGCATGATTTTAATGAAGC	lldP-F
6	GCACAAAGACAAAACCACAAACC	lldP-R
7	CTTAGCAGGAGACATTCCTTCC	kan_upstream_out
8	GGATCAAGCCTGATTGGGAG	kan_downstream_out
9	GGTCAATCGAGAATATCGTCAACTG	ermB-F
10	GCCAGTTTCGTCGTTAAATGC	ermB-R
11	CCTGCAGGTCGACTCTAGAGCCTGCAGGTCGACTCTAGAG	P_cad_::lctP_fwd
12	CATTAGAATAGGCGCGCCTGTTAGAATATTAACGTTAGTATAAACG	P_cad_::lctP_rev
13	CCTGCAGGTCGACTCTAGAGTATCGATAACAAAATGCTATAGC	P_cad_::lldP_fwd
14	CATTAGAATAGGCGCGCCTGTTATAATAATGACAAGATGAAAGTC	P_cad_::lldP_rev
15	CCTGCAGGTCGACTCTAGAGTAAATCAAACGTAGGAGGC	P_cad_::lrgAB_fwd
16	CATTAGAATAGGCGCGCCTGTTAGAAGAATATTGCTACAAAGAC	P_cad_::lrgAB_rev
17	aacgaactaacgtaaggtgg	13955 F
18	tgtctgaacctgcaggtcgacattagggaagtaggaatagttatgaag	P_cad_::13955_fwd
19	agctcggtacccggggatccttatttttctattgctttatcatcactaacaattaaag	P_cad_::13955_rev
20	GGCGGCCGCTGCATGCaaactggataaattcaaatgaataattaatgatg	ML213
21	CTTTGCTCCCGGGCATATGaactattcctacttccctaattaattttttaaaac	ML214

### Generation of 3-fluoropyruvic acid-resistant *S. aureus* mutants

From a 5 mL overnight culture of *S. aureus* JE2 grown in TSBG, 1 mL was centrifuged and washed twice with phosphate-buffered saline (PBS) and diluted to an OD_600_ of 1 in PBS. This was used as the starting inoculum for the lawn of bacteria swabbed onto TSA. A sterile 6 mm diameter blank disk was embedded with 15 µL 110 mM 3-fluoropyruvic acid sodium salt monohydrate (Santa Cruz Biotechnology) and placed in the center of the plate. The plates were placed at 37°C for 24 hours and photographed. Resistant colonies selected in the zone of inhibition were screened for resistance to the toxic analog in TSBG containing 3 mM 3-FP. In the secondary screen, overnight cultures of the *lctP/lldP* or Δ3 mutant were swabbed onto TSA plates where a sterile 6 mm diameter blank disk was embedded with 15 µL 110 mM 3-fluoropyruvic acid sodium salt monohydrate (Santa Cruz Biotechnology) and placed in the center of the plate. The plates were placed at 37°C for 24 hours and photographed. Overnight cultures of colonies isolated from the zone of inhibition were swabbed on a TSA plate with a disk embedded with 15 µL 110 mM 3-fluoropyruvic acid sodium salt monohydrate (Santa Cruz Biotechnology) placed in the center of the plate to confirm resistance.

### Extracellular pyruvate and lactate analysis

Supernatant samples were collected from bacterial cultures grown in TSBG with 0.5 mM pyruvate every 2 h for a total of 6 h or from the wells of a 96-well plate grown in the Tecan for 24 hours with CDM, CDMG, CDMP, and CDML. The bacteria were pelleted, and the supernatant was collected in a clean 1.5 mL tube, and samples were frozen at −20°C. The pyruvate assay was performed as previously described (42). Briefly, samples were added to a black 96-well plate with a clear bottom and brought to 50 µL total volume with the pyruvate assay buffer (100 mM potassium phosphate, 1 mM EDTA, and 1 mM MgCl_2_). Then, 150 µL of an enzyme mix containing 10 mM flavin adenine dinucleotide, 0.2 mM thiamine pyrophosphate, 0.2 U/mL pyruvate oxidase, 0.2 U/mL horseradish peroxidase, and 50 mM Amplex Red in the assay buffer was added to each well and mixed by pipetting. The plate was incubated for 15 minutes at room temperature in the dark, and then the OD_570_ was measured using a Tecan microplate reader. A standard curve from 0 to 10 mM was generated and used to calculate the amount of pyruvate per sample.

The amount of lactate present in the 24-hour Tecan-grown samples was measured using the Enzytec Liquid D-/L-Lactic acid test kit (R-biopharm, catalog no. E8240) per the manufacturer’s instructions. Briefly, 10 µL of culture supernatant was added to the wells of a 96-well microtiter plate, followed by the addition of 250 µL master mix (200 µL reagent 1 and 50 µL reagent 2). After 15 minutes incubation, at room temperature, the plate is read at 340 nm, and the amount of lactate is calculated using a linear regression of a standard curve from 10 mM to 0.3125 mM.

### Whole-genome sequencing

Genomic DNA of the JE2 3-FP-resistant strains was isolated utilizing the Wizard Genomic DNA Purification Kit (Progmega Corp). The DNA samples were then quantified with a Qubit (Thermo), and 1 ng was tagmented, and Nextera Indexes were added per the Nextera XT DNA Library Prep kit (Illumina) and associated protocols. Libraries were validated by running 5 µL of PCR cleanup on a 1% agarose gel, then bead-normalized and pooled in equal volumes. Pooled normalized libraries (2 nM starting concentration assumed) and PhiX were diluted and denatured according to the MiSeq Systems User Guide, with a final concentration of 80 pM. The final pool was heated at 96°C for 3 minutes to ensure denaturation before sequencing on a MiSeq using read length 2 × 300 bp onboard fastq file generation and sample demultiplexing, generating 0.6–1.4 million paired reads per sample. Reads were processed using CLC Genomics Workbench (v. 20.0.4) and the Microbial Genomics Module (v. 20.1.1) “Type a known species” workflow, where reads were mapped to the JE2 genome to identify single and multi-nucleotide variants.

### Radiolabeled pyruvate transport assay

Overnight cultures of single colonies inoculated into 5 mL of TSBG were used to start 25 mL TSBG with 0.5 mM pyruvate cultures in 250 mL flasks (250 rpm) at an OD_600_ = 0.05. Cells were grown for 4 hours. The entire culture (25 mL) was centrifuged at 4,000 rpm in the Sorvall Legend X1R Centrifuge (Thermo Scientific) for 10 minutes. The supernatant was removed, and cells were resuspended in TSBG to an OD_600_ = 10. Once the cell suspension was prepared, 100 µL were aliquoted into microcentrifuge tubes and placed on ice. For the assay, 900 µL of pre-warmed (37°) TSBG was added to 100 µL OD_600_ = 10 cells for a final OD_600_ = 1 (~10^8^ CFU). For the blank, 100 µL of the sample was collected on a 25 mM diameter 0.45 µM nitrocellulose membrane (Whatman) and washed with 10 mL PBS with 10 mM pyruvate. The remaining 900 µL of the sample was added to pre-warmed 5 mM ^14^C-pyruvate to give a final concentration of 0.5 mM ^14^C-pyruvate for the assay and incubated at 37°C. For the competition assays, 50 mM unlabeled pyruvate or lactate was added to the 5 mM ^14^C-pyruvate stock pot to result in 10× (5 mM unlabeled competitor). Samples (100 µL) were collected at timed intervals (e.g., 30 seconds, 1 minute, 2 minutes, 4 minutes, 6 minutes, 8 minutes, and 10 minutes) after adding the radiolabeled pyruvate to the bacteria. All samples were immediately applied to a 25 mM diameter 0.45 µM nitrocellulose membrane with a vacuum and washed with 10 mL PBS containing 10 mM pyruvate. Bacteria on the washed filters were placed into scintillation tubes and allowed to dry for at least 16 hours. Filter count scintillation fluid (Perkin Elmer) was added to the samples. The filters were allowed to dissolve for over 16 hours in the scintillation fluid and then counted using a Packard Tri-Carb 2910TR scintillation counter to determine the DPM per sample. The transport assay curves were fit by non-linear regression using a first-order equation (Pyr = A*(1-exp(-B*Time))). The given equation models the transport of pyruvate over time. In this equation, Pyr represents the amount of pyruvate transported, A denotes the maximum capacity or asymptote of pyruvate transport, B is the rate constant governing the transport process, and Time refers to the duration of transport. The equation describes a saturation curve, where pyruvate transport begins rapidly and eventually slows down as the transport system becomes saturated. Non-linear regression was employed to fit this equation to the transport assay curves, adjusting parameters A and B to accurately match the shape of the experimental data. Specifically, the term exp(-B*Time) calculates the fraction of pyruvate left not transported at any given time, taking into account the decay rate dictated by parameter B. By subtracting this fraction from 1 and multiplying it by the maximum capacity A, the model estimates the absolute quantity of pyruvate transported, thereby capturing the dynamics of pyruvate uptake over time.

### Statistical analysis

All graphs and statistical analysis were generated in Prism GraphPad.

## SUPPLEMENTAL MATERIAL

Supplemental figure and table jb.00163-25-s0001.docx Figure S1 and Table S1.

ASM does not own the copyrights to Supplemental Material that may be linked to, or accessed through, an article. The authors have granted ASM a non-exclusive, world-wide license to publish the Supplemental Material files. Please contact the corresponding author directly for reuse.
