# Supplementary material for: Staphylococcus aureus encodes four differentially regulated pyruvate transporters
Source: J Bacteriol. 2025 Oct 10;207(11):e00163-25. doi: 10.1128/jb.00163-25 (PMC12632267; doi:10.1128/jb.00163-25)
Supplement: Supplemental figure and table — Figure S1 and Table S1. [file jb.00163-25-s0001.docx]

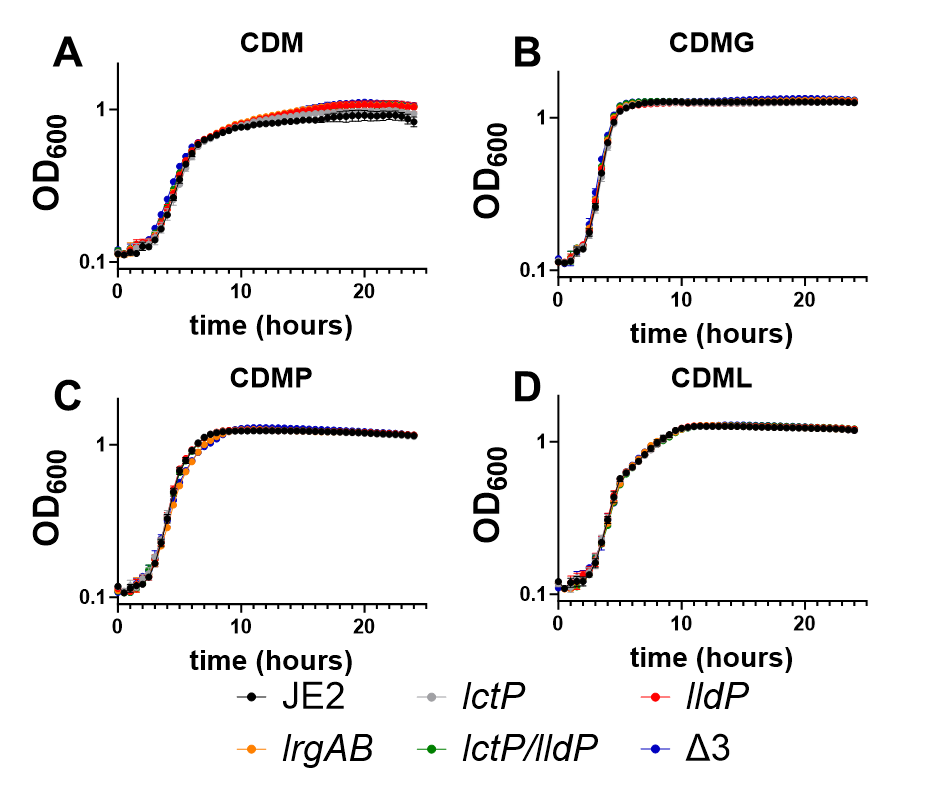


**Supplemental Figure 1. No growth defect of the Δ3 mutant in media supplemented with pyruvate or lactate.** Aerobic growth analysis of JE2 and isogenic mutants in A) CDM, B) CDM with 14 mM glucose (CDMG), C) CDM with 28 mM pyruvate (CDMP), and D) CDM with 28 mM lactate (CDML). Data represent the mean ± SD from three biological experiments, repeated 3 times.

**Supplemental Table 1. Zones of inhibition caused by the toxic pyruvate analog 3-Fluropyruvic acid on TSAG plates of the *lctP lldP* and Δ3 mutant.**

| **Strain** | **Zone of complete clearing (cm)** | **zone of haziness (cm)** |
| --- | --- | --- |
| JE2 | 3.5 | 0 |
| *lctP* | 3.175 | 2.22 |
| *lldP* | 3.5 | 2.9 |
| *lctP/lldP* | 2.9 | 1.27 |
| Δ3 | 0 | 2.9 |
